# The impact of chemicals and additives on maize physiology and resistance to *Spodoptera frugiperda*

**DOI:** 10.3389/fpls.2026.1767144

**Published:** 2026-01-28

**Authors:** Abdul Basit, Muhammad Humza, Tong-Xian Liu, Youming Hou

**Affiliations:** 1 State Key Laboratory of Ecological Pest Control for Fujian and Taiwan Crops, Key Laboratory of Biopesticides and Chemical Biology, MOE, College of Plant Protection, Fujian Agriculture and Forestry University, Fuzhou, China; 2 Guizhou Provincial Key Laboratory for Agricultural Pest Management of the Mountainous Region, Institute of Entomology, and Institute of Plant Health and Medicine, Guizhou University, Guiyang, China; 3 State Key Laboratory of Wheat Improvement, Shandong Provincial Key Laboratory of Agricultural Microbiology, College of Plant Protection, Shandong Agricultural University, Tai’an, China

**Keywords:** additives, maize, pest management, photosynthesis, physiological responses, *Spodoptera frugiperda*

## Abstract

**Background:**

*Spodoptera frugiperda* is a highly invasive pest that significantly threatens maize production globally. This work aims to report the physiological and biochemical impacts of different chemical treatments (DMSO, methyl oleate, DMF, xylene, uniconazole, azadirachtin, and chlorantraniliprole) on maize photosynthetic capacity and resistance response mechanisms from *S. frugiperda*.

**Results:**

We found a dose‐dependent effect on maize photosynthesis; lower concentrations of these chemicals promoted photosynthetic rate, whereas higher concentrations inhibited photosynthesis, especially in lower leaves. Mortality bioassays proved the dose-related response to the toxic potential of DMSO, DMF and xylene. However, the Y-tube bioassay revealed no remarkable changes in olfactory responses, thus indicating that the observed mortality was largely contributed by direct chemical toxicity rather than behavioral alterations. At the molecular level, cytochrome P450 genes *(Sf CYP6AB12, Sf CYP6AE43, Sf CYP9A58* and *Sf CYP9A59*) were significantly overexpressed by chlorantraniliprole, and they were considered to be resistant genes against insecticides. Likewise, other compounds such as azadirachtin and uniconazole also selectively affected some P450 genes, providing additional evidence of the involvement of P450s in *S. frugiperda* metabolic resistance.

**Conclusions:**

These results demonstrate the involvement of P450s in the development of insecticide resistance and suggest the importance of chemical dose on control of insect pests.

## 1 Background

Maize (*Zea mays* L.), one of the most widely cultivated cereal crops in the world, is China’s second most important crop after rice and wheat (Willer and Lernoud, 2017; Luo et al., 2023). Maize production and consumption in China rank second in the world, and the northeast region possesses most of the production (Liang et al., 2021; Pei and Wu, 2023). The fall armyworm, *Spodoptera frugiperda* (Smith), an extremely invasive and polyphagous pest native to the Americas, primarily feeds on and infests maize (Siebert et al., 2012; Devi et al., 2024). Therefore, the quick spread of *S. frugiperda*, especially in China, poses a serious threat to agricultural production and national food security (Siebert et al., 2012; Yeboah et al., 2021). Larvae of *S. frugiperda* frequently feed on young leaves, leaf whorls, and tassels and result in massive decreases of yield if the pest is not managed (Cook et al., 2007; Chimweta et al., 2020; Bengyella et al., 2021; Toepfer et al., 2021).

Plant-insect herbivore interactions are among the most common and ubiquitous ecological interactions (Schoonhoven et al., 2005). Considerable effects on community structure, primary productivity, and even global biosphere-atmosphere processes via their effects on plant distribution (as pests), abundance, and evolutionary trajectories (Belovsky and Slade, 2000; Karl et al., 2008; Metcalfe et al., 2014). By physically consuming leaf material, insect herbivores have a direct negative effect on the process of photosynthesis and, therefore, the plant’s energetic output. This loss of most of the layers brings about changes in both core (respiration) as well as secondary metabolic pathways (synthesis of defensive compounds), all of which ultimately can impact the nutritional composition of the entire plant and influence its toxicity (Herms and Mattson, 1992; Kerchev et al., 2012). In addition to the specific ones in response to herbivory, plants can also release volatile organic compounds (VOCs) as chemical signals or defenses (Rowen and Kaplan, 2016). This induced change is systemic, influencing not only undamaged tissue but also the general physiological status of the plants, affecting their growth and defense (Agrawal, 2000; Staudt and Lhoutellier, 2007; Wu and Baldwin, 2009). Leaf damage due to herbivory often activates the upregulation of defense-related genes while suppressing genes related to photosynthesis (Bilgin et al., 2010). However, the resulting impacts on photosynthetic efficiency are complex, and whilst previous studies have reported increased rates of photosynthesis—typically interpreted as a compensatory response—others have recorded decreases in photosynthetic performance, depending on the specific context and degree of herbivory (Zangerl et al., 2002; Nykänen and Koricheva, 2004; Nabity et al., 2009).

Among the various pest control methods, synthetic insecticides have been identified as an effective tool against *S. frugiperda* infestations, largely owing to their high efficiency and commendable cost-effectiveness, despite their non-target effects on beneficial insects (Desneux et al., 2007; Susanto et al., 2021). Specifically, the Chinese Ministry of Agriculture and Rural Affairs officially suggested the usage of emamectin benzoate, chlorantraniliprole, and spinosad as effective chemical controls for *S. frugiperda* in 2020 (Zhang et al., 2020; Li et al., 2021; WAN et al., 2021). However, these chemical insecticides degrade into sublethal concentrations that induce a range of effects on targeted pests, including behavioral changes, resistance development, hormesis effects, and physiological disruption (Ullah et al., 2024).

By 2017, resistance to around 29 different insecticidal compounds was already reported in *S. frugiperda* from the U.S, predominantly due to over-reliance on these chemicals (WAN et al., 2021). Moreover, the resistance of some *S. frugiperda* populations to organophosphate and pyrethroid insecticides in China has posed more challenges to pest control (Zhang et al., 2020).

The phenomenon of the development of insecticide resistance in insects is so complicated that the use of various synergists can be employed to evaluate their effectiveness under different environmental conditions, especially as they are known to form part of the resistance mechanism (Mohan and Gujar, 2003). There are many studies on cytochrome P450 monooxygenases (P450s) associated with ` resistance (Ullah et al., 2024; Jiang et al., 2023; Liu et al., 2024). These enzymes are important for insecticide detoxification and are members of well-known families underlying adaptation to insecticides (Dermauw et al., 2013). Recent comparative genomic studies have shown a remarkable expansion of the cytochrome P450 gene family in FAW, comprising a total of 425 P450 genes, including 283 FAW-specific P450 genes (Gui et al., 2022). In addition, performing qRT-PCR analysis on cytochrome P450 genes in field FAW populations may be useful for the characterization of resistance mechanisms (Lao et al., 2015). The over-expression of some P450 genes is suggested as one of the major factors associated with elevating the P450 activity of FAW, which might provide resistance to insecticides like chlorantraniliprole (Gong et al., 2013; Elzaki et al., 2016). Transcriptomic and genomic analysis of the FAW suggested that 117–425 P450 genes, such as *CYP321A8*, *CYP321A9*, and *CYP321B1* may have important functions in insecticide detoxification and its resistance (Palli and Chen, 2021; Zhang, 2021).

The current research was aimed at evaluating the impact of additives [dimethyl sulfoxide (DMSO), methyl oleate, dimethyl formamide (DMF), and xylene) and chemical insecticides (uniconazole, azadirachtin, and chlorantraniliprole) on the physiological responses of maize and *S. frugiperda.* Photosynthetic efficiency was determined by measuring CO_2_ assimilation and chlorophyll fluorescence, as well as plant morphophysiological traits such as height and biomass production. Mortality bioassays were performed to assess the insecticidal efficacy. The Y-tube olfactory assays were used to unravel pest behavioral responses to additives and insecticides. This overall approach links plant health, toxicity, and insect behavior to better refine sustainable pest management strategies.

## 2 Methods

### 2.1 Insect and plant culture

Maize (*Z. mays* L., Cultivar: Heyu 808, Jinqiannonghye Co. Ltd.) was cultivated under controlled conditions (25 ± 2 °C, 60–70% relative humidity, 16:8 h light: dark photoperiod). For uniformity, seeds were germinated in sterilized soil spots from surface-stabilized seeds. *Spodoptera frugiperda* colonies were maintained in the laboratory at standard conditions (27 ± 1 °C, 65 ± 5% relative humidity), with larvae reared individually on an artificial diet to prevent cannibalism. Adult moths were kept in mating cages and provided with a 10% honey solution to facilitate oviposition on maize leaves. This system closely regulated all relevant environmental factors, minimizing variability and ensuring the physiological integrity of both the host plant and insect for bioassays.

### 2.2 Preparation of solutions

The additives DMSO, methyl oleate, DMF, and xylene, along with the insecticides uniconazole, azadirachtin, and chlorantraniliprole, were purchased from a local supplier and stored under optimal conditions to maintain stability. Stock solutions were prepared by dissolving each compound in a suitable solvent and diluting to the desired concentrations: DMSO, methyl oleate, and xylene were used at concentrations of 10%, 20%, and 40%, while DMF was prepared at 5%, 10%, and 20%. For the insecticides, azadirachtin was tested at concentrations of 10, 20, and 40 mg/l; chlorantraniliprole at 150, 300, and 600 mg/l; and uniconazole at 0.6, 3, and 15 mg/l (Basit et al., 2025). Equivalent volumes of hexane were used as a solvent control in all treatments to enable organomimetic testing and to account for any solvent-related effects during insect response analysis.

### 2.3 Photosynthesis evaluation

Healthy maize leaves were selected for evaluation and treated with different concentrations of the prospective chemicals. These leaves were cultivated in a controlled environment growth chamber at 25 ± 2 °C, 60–70% relative humidity (RH), and a 16:8 light: dark photoperiod. Photosynthetic rates were measured using a Li-6400XT Portable Photosynthesis System, which records leaf gas exchange in terms of CO_2_ assimilation.

Experiments were conducted at various initial light intensities, ranging from 400 µmol m^-2^ s^-1^ to 2,000 µmol m^-2^ s^-1^, in order to generate light response curves and assess the photosynthetic efficiency of the plants. Leaf temperature was maintained at 25 °C, relative humidity at 68%, and atmospheric CO_2_ concentration at a constant 400 ppm.

For uniformity and to capture representative data, fresh, fully expanded leaves from different positions on the plant (e.g., leaf positions 1, 3, 5, etc.) were selected. The photosynthetic rate was calculated as the average value over 5 minutes at each light intensity, reported in µmol CO_2_ m^-2^ s^-1^.

### 2.4 Effects of chemical treatments on plant growth and physiological traits

To assess the effects of various chemical treatments on plant growth and physiological traits, maize plants were grown under controlled conditions at 25 ± 2 °C, 60–70% relative humidity (RH), and a 16:8 h light: dark photoperiod. Different concentrations of the chemicals (additives and insecticides) (DMSO, methyl oleate, DMF, xylene, uniconazole, azadirachtin, and chlorantraniliprole) were applied to the plants, while an untreated control group was maintained for comparison.

The following growth parameters were measured after the treatment: plant height, number of leaves, fresh weight, dry weight, and total chlorophyll content. Total chlorophyll content was assessed using a SPAD-502 Chlorophyll Meter. Plant height was recorded using a digital caliper, while both fresh and dry weights were measured using a precision digital balance (Ohaus Scout).

### 2.5 Toxicological effects of chemical treatments on *S. frugiperda* mortality

To evaluate the toxicological effects of different chemical treatments (DMSO, methyl oleate, and xylene were used at concentrations of 10%, 20%, and 40%, while DMF was prepared at 5%, 10%, and 20%) on *S. frugiperda* mortality, a laboratory bioassay was conducted. A control group (water) was maintained without chemicals. The chemicals were incorporated into the larvae’s diet to ensure consistent exposure. The larvae were reared under constant conditions of temperature, humidity, and photoperiod to avoid the influence of environmental variability. Mortality was assessed at 24-, 48-, and 72-hours post-exposure by gently prodding the larvae with a fine brush. Larvae that were motionless were recorded as dead, and those that were severely paralyzed or unresponsive (moribund) were recorded separately. Any visible abnormalities, such as molting failure or deformities, were also documented. Mortality rates were calculated as a percentage of the total number of exposed larvae in each treatment group compared to the control group.

### 2.6 Enzyme activity

Eighty third instar *S. frugiperda* larvae were also exposed to the highest concentration of each of the compounds tested, compared with as control treated sample. The larvae were weighed (soaked with liquid), homogenized, and rinsed with a 0.05 M sodium phosphate buffer (pH 7.3) at room temperature. After homogenization, samples were centrifuged at 19538 × g for 10 min at 4 °C. The supernatant was decanted and transferred into new tubes and again centrifuged at 19538 × g for 15 min. The ultimate supernatants were used for the measurements of enzyme activities. The enzymatic assays for AChE, GST, CarE, and P450 enzymes were conducted according to the manufacturer’s instructions. AChE activity was recorded by 412 nm absorbance, whereas 450 nm recorded the GST, CarE, and P450 activities. Protein concentration of the samples was determined using a protein quantification kit following the manufacturer’s protocol and was expressed using BSA as a standard.

### 2.7 Olfactory preference assay using Y-tube olfactometer

The olfactory preferences of gravid female *S. frugiperda* towards various chemical treatments were assessed using a Y-tube olfactometer, a dual-choice device that quantifies insect attraction, repulsion, or neutrality to specific chemicals. The chemicals tested included DMSO, methyl oleate, DMF, xylene, uniconazole, azadirachtin, and chlorantraniliprole, applied at different concentrations (mentioned previously). Insects were introduced into the central arm of the Y-tube, where they encountered two diverging airflow streams; one containing the test chemical stimulus and the other a neutral control (hexane). A constant airflow (500 ml/min) was maintained during the experiment, ensuring uniform diffusion of the chemical while minimizing stochastic effects. A definitive response was recorded when the insect crossed a designated demarcation point in either arm of the Y-tube. Non-responding insects were classified as no choice. To maintain experimental consistency, strict environmental conditions, including temperature, humidity, airflow velocity, and photoperiod, were rigorously controlled. Exhaustive decontamination procedures were followed between replications to prevent olfactory residue buildup.

### 2.8 Extraction of RNA and cDNA synthesis

Total RNA was extracted from 3^rd^ instar larvae of *S. frugiperda* at maximum concentration of each compound of chemical with the Eastep Super Total RNA Kit (LS1040) (Promega, Madison, WI, USA) according to the manufacturer’s instructions. RNA quality was checked by agarose gel electrophoresis and purity was measured with a NanoDrop 2000 spectrophotometer (Thermo Fisher Scientific). First-strand cDNA was then synthesized using the PrimeScript 1st-Strand cDNA Synthesis Kit (6110A) (Takara Bio, Beijing, China) according to the manufacturer’s instructions. The resulting cDNA was kept at ­20 °C for subsequent usage.

### 2.9 Quantitative reverse transcription polymerase chain reaction analysis

The relative expression of key genes involved in insecticide resistance was assessed by RT-qPCR from 3^rd^ instar larvae of the *S. frugiperda* after feeding at the maximum concentration of each chemical. *Sf RYR, Sf CYP6AB12, Sf CYP6AE43, Sf CYP6AN4, Sf CYP9A58, Sf CYP9A59*. The reference gene used for normalization was EF1α. Primer sequences for RT-qPCR amplifications are provided in **Supplementary Table S1**. RT-qPCR was performed using the ABI 7500 Real-Time PCR System (Applied Biosystems, USA) with a 20 µL reaction mixture, consisting of 10 µL of 2× SYBR^®^ Premix Ex Taq (Takara, Dalian, China), 0.5 µL of each 10 µM forward and reverse primer, 2 µL of 10-fold diluted cDNA template, and 7 µL of ddH_2_O. The thermal cycling conditions included an initial preheating at 95 °C for 30 s, followed by 40 cycles of denaturation at 95 °C for 30 s, annealing at 60 °C for 40 s, and elongation at 72 °C for 60 s. Three biological replicates were used for each treatment, with three technical replicates for each sample. The primer sequence is shown in the **Supplementary Table S1**.

### 2.10 Statistical analysis

Data from the different treatments of experiments were analyzed by means of statistical analysis according to the method for each assay. The treatment group was compared to the controls using one-way ANOVA (Analysis of Variance) followed by Tukey’s multiple comparison test for *post-hoc* analysis when the difference was significant. Statistically significant mortality rate, enzyme activity, and photosynthesis rate were tested at different concentrations of the chemical treatments. The expression levels of the gene were determined by RT-qPCR and were analyzed with ANOVA, and the fold change in relative gene expression was calculated using the 2−ΔΔCt method, where ΔCt is the difference between the threshold cycle values of the target gene and the reference gene (EF1α). In all experiments, a p < 0.05 was considered statistically significant. All measurements of data are expressed as means ± SEM and analyzed using SPSS or GraphPad Prism software (IBM, USA; GraphPad Software, USA, respectively).

## 3 Results

### 3.1 Effects of additive treatment on photosynthetic rate

**Figure 1:**
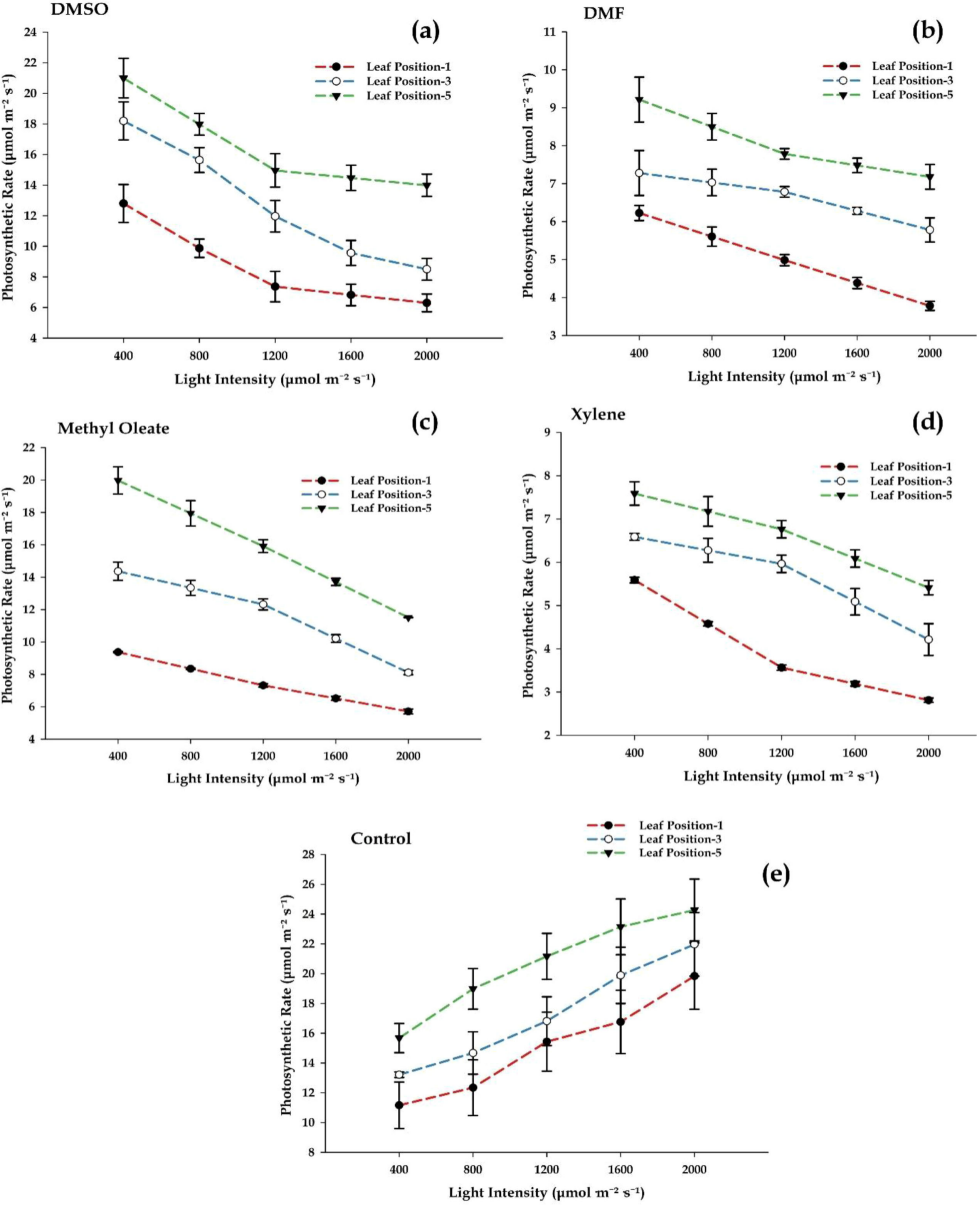
Impact of chemical treatments on photosynthetic rate across different leaf positions and light intensities. Photosynthetic rate (µmol m^-2^ s^-1^) was measured under increasing light intensities (µmol m^-2^ s^-1^) for three leaf positions: Lower (Position-1, red), Middle (Position-3, blue), and Upper (Position-5, green). **(a–d)** Additives treatments [dimethyl sulfoxide (DMSO), methyl oleate, dimethyl formamide (DMF) and xylene] caused a decline in photosynthesis, with xylene and DMF showing the strongest inhibition. **(e)** Control plants exhibited a natural increase in photosynthetic rate with light, highlighting the disruptive effects of chemical exposure. Error bars represent standard deviations (SD).

The photosynthetic rate exhibited varying responses to chemical treatments depending on leaf position and light intensity. Treatment with DMSO resulted in a significant reduction in photosynthetic rate as light intensity increased. The highest photosynthetic rate was observed in leaf position-5, while position-3 showed intermediate values and position-1 had the lowest. Notably, the decrease in photosynthetic rate was most pronounced in position-1, indicating that lower leaves were more sensitive to DMSO compared to higher leaves. DMSO, a common additive, appears to disrupt normal photosynthetic function, particularly in lower leaf positions (**Figure 1a**). The effects of DMF were more pronounced than DMSO. Photosynthetic rates consistently decreased as light intensity increased, with overall rates being lower compared to DMSO treatment. Leaf position-5 continued to exhibit the highest photosynthetic rate, but position-1 showed the most significant decline. Interestingly, the difference between leaf position-3 and leaf position-5 was minimal, suggesting that DMF exerts a more uniform inhibitory effect across all leaf positions, significantly diminishing photosynthetic activity. These findings suggest that DMF is a more potent disruptor of photosynthetic processes compared to DMSO (**Figure 1b**). Methyl oleate caused a reduction in the rate of photosynthesis, although the effect was milder compared to DMF and DMSO. A gradual decrease in photosynthetic rate was observed as light intensity increased, with position-5 maintaining the highest rate and position-1 displaying the lowest. These results indicate that methyl oleate has some inhibitory effects on photosynthesis, but these effects are less severe than those observed with DMF (**Figure 1c**). Xylene had the most substantial inhibitory effect on photosynthesis. Photosynthetic rates decreased sharply with increasing light intensity, with position-1 showing the greatest reduction, reaching values below 4 µmol m^−2^ s^−1^ at the highest light intensities. Even position-5, typically associated with the highest net photosynthesis rate, displayed a significant decrease. These results indicate that xylene profoundly impairs photosynthetic processes, potentially through disruption of chloroplast function or gas exchange pathways (**Figure 1d**). In contrast, the control group (water) exhibited a typical response, with photosynthetic rates gradually increasing in accordance with light intensity. The highest photosynthetic rate was observed in position-5, followed by position-3 and position-1. Under control conditions, plants performed optimal photosynthesis, demonstrating the natural, uninhibited process of photosynthesis (**Figure 1e**). The data from this study highlight the inhibitory effects of various chemical treatments on photosynthetic rate. The results demonstrate a clear dose- and leaf position-dependent response, with DMSO and DMF showing the most significant disruptions to photosynthetic function, followed by xylene, and finally methyl oleate, which exhibited the mildest effect. These findings underscore the potential impact of chemical additives on plant physiology, especially in relation to photosynthesis.

### 3.2 Effects of insecticides treatments on photosynthetic rate

**Figure 2:**
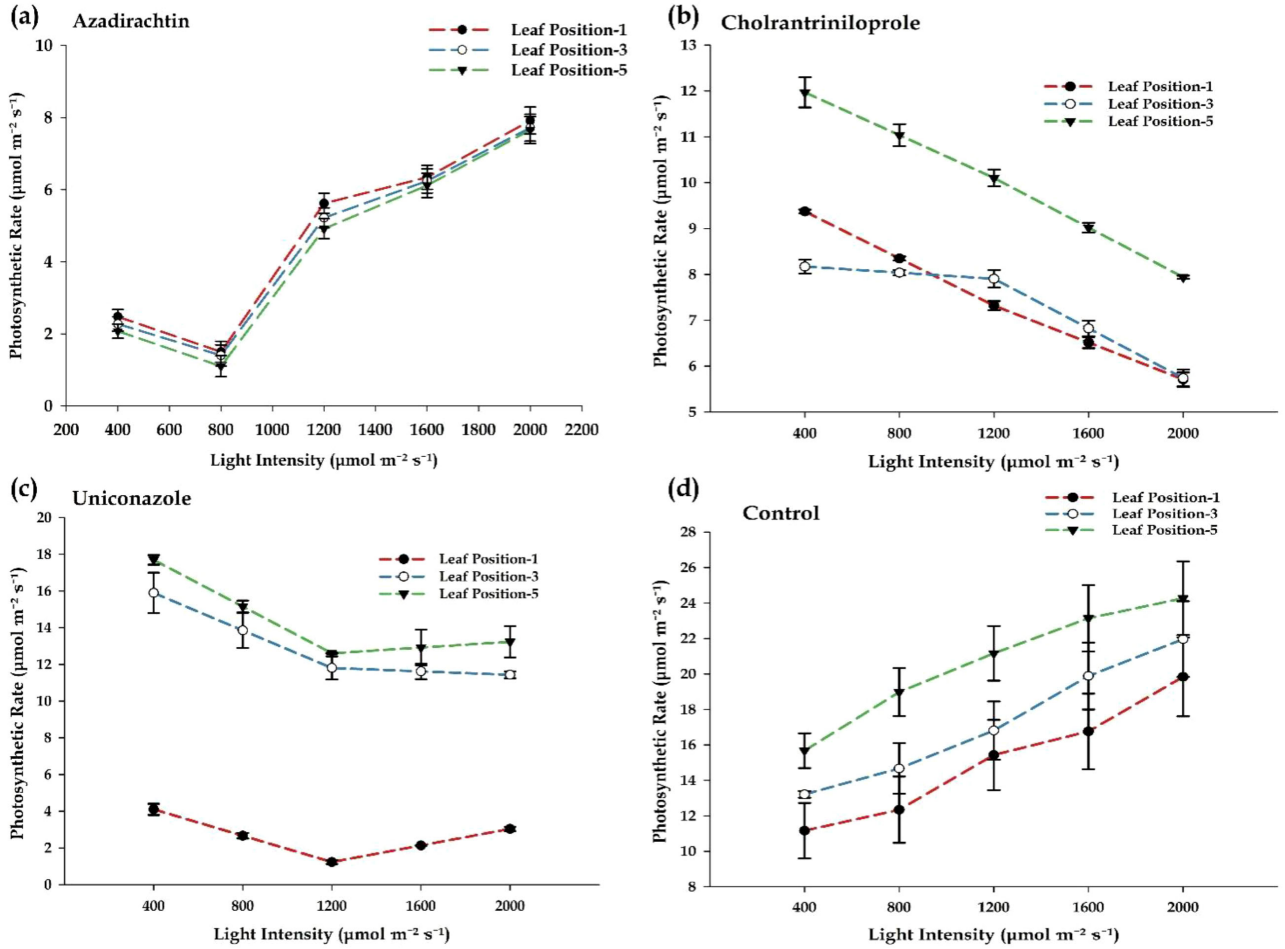
Effect of different chemical treatments on photosynthetic rate across varying light intensities and leaf positions. **(a)** Azadirachtin showed an initial low photosynthetic rate, followed by a sharp increase at higher light intensities. **(b)** Chlorantraniliprole caused a steady decline in photosynthesis, with lower leaf positions being most affected. **(c)** Uniconazole led to a severe reduction in photosynthesis, particularly in lower leaves. **(d)** Control plants exhibited a natural increase in photosynthetic rate with rising light intensity, highlighting the inhibitory effects of chemical treatments. Error bars represent standard deviations (SD).

The effects of different insecticide treatments on photosynthetic rate at varying light intensities and leaf positions are presented in **Figure 2**. The application of azadirachtin resulted in minimal changes to photosynthetic rate at low light intensities, with no significant decrease observed. However, at light intensities greater than 800 µmol m^-2^ s^-1^, the photosynthetic rate notably increased across all leaf positions. This response suggests that azadirachtin does not strongly suppress photosynthesis and may potentially enhance the photosynthetic light response at higher light intensities. These results indicate a facilitative rather than inhibitory role of azadirachtin in photosynthesis, particularly at higher light levels (**Figure 2a**). Chlorantraniliprole had a marked inhibitory effect on photosynthetic activity. As light intensity increased, the photosynthetic rate consistently declined, with lower leaf positions experiencing a greater reduction. Leaf position-1 showed the steepest decrease in photosynthetic rate, while leaf position-5 maintained relatively higher rates. However, the general downward trend suggests that chlorantraniliprole negatively affects photosynthesis across all leaf positions, with the most significant impact on the lower leaves of the plant canopy (**Figure 2b**). The effects of uniconazole displayed a dual trend. Middle and upper leaf positions (positions-3 and -5) showed a gradual decrease in photosynthetic rate as light intensity increased, while the rate in leaf position-1 dropped sharply at all light intensities. This indicates that uniconazole has a more pronounced effect on lower canopy leaves, possibly due to disruptions in stomatal conductance or chlorophyll synthesis. These results suggest that uniconazole may interfere with photosynthetic processes, particularly in the lower portions of the plant, where its impact is most severe (**Figure 2c**). In contrast, the control group (water) exhibited the expected increase in photosynthetic rate as light intensity rose. This response followed a natural pattern, with the highest rates observed in the upper canopy leaves (position-5), followed by the middle (position-3) and lower (position-1) leaves. The control group demonstrated typical photosynthetic behavior, with optimal rates achieved under natural light conditions (**Figure 2d**). The results indicate that different insecticide treatments have distinct effects on photosynthetic rate depending on light intensity and leaf position. Azadirachtin seems to enhance photosynthesis at higher light intensities, while chlorantraniliprole and uniconazole both inhibit photosynthetic activity, with uniconazole showing a stronger impact on the lower leaves of the plant. These findings suggest that the effects of chemical treatments on plant physiology can vary significantly based on environmental conditions and leaf position within the plant canopy.

### 3.3 Impact of chemical (additives and insecticides) treatments on plant growth and physiological traits

The effects of various chemical treatments on plant growth and physiological traits are illustrated in **Supplementary Figure S1**. All chemical treatments resulted in significant reductions in key plant parameters such as total chlorophyll content, plant height, number of leaves, fresh weight, and dry weight (p < 0.01), when compared to the control group, which exhibited optimal growth. This reduction in plant health and productivity can be attributed to the stress induced by chemical exposure, which hampers overall plant physiological functions. Chlorantraniliprole was among the most impactful chemicals, showing marked reductions in total chlorophyll content, plant height, and biomass accumulation. The treated plants also exhibited a significant decrease in leaf number, indicating a strong disruption in leaf development. This suggests that chlorantraniliprole may interfere with hormonal regulation or cell division, leading to impaired plant growth and reduced productivity (**Supplementary Figure S1a**). Azadirachtin treatment also led to significant reductions in chlorophyll content, plant height, and biomass accumulation. Both fresh and dry weights of the treated plants were significantly lower, indicating a substantial decrease in the plant’s ability to accumulate biomass. These findings suggest that azadirachtin has a detrimental impact on plant health, affecting both photosynthetic efficiency and growth (**Supplementary Figure S1b**). Uniconazole caused one of the most severe reductions in plant height and biomass production. The treated plants remained notably stunted in comparison to the control group, suggesting that uniconazole strongly inhibits plant elongation and metabolic activity. These effects may be linked to the chemical’s interactions with hormonal pathways involved in plant growth and elongation (**Supplementary Figure S1c**). Both DMSO and DMF resulted in lower chlorophyll content, shorter plant height, and inhibited biomass production. The number of leaves was consistently lower in treated plants compared to the control group, indicating that these chemicals negatively affected leaf expansion and overall vegetative growth. These results suggest that DMSO and DMF may disrupt photosynthetic processes and other metabolic pathways critical for plant development (**Supplementary Figures S1d, e**). Methyl oleate caused a moderate reduction in plant health, with a slight decrease in chlorophyll content and biomass when compared to control plants. While the treated plants showed elevated chlorophyll content and biomass relative to other chemicals, the overall values remained significantly lower than the control group. This suggests that methyl oleate has a mild but still inhibitory effect on plant physiological functions (**Supplementary Figure S1f**). Xylene was one of the most aggressive inhibitors of plant growth, leading to drastic reductions in all measured parameters. The most significant reduction was observed in fresh weight, indicating that xylene may interfere with water uptake, nutrient assimilation, and/or photosynthetic efficiency. The overall plant health was severely compromised, highlighting the potent toxicity of xylene on plant physiological functions (**Supplementary Figure S1g**). The chemical treatments applied in this study consistently resulted in detrimental effects on plant growth and physiological functions. Chlorantraniliprole, uniconazole, and xylene were the most harmful, significantly reducing key parameters such as chlorophyll content, plant height, and biomass. DMSO, DMF, and methyl oleate also inhibited plant growth, but to a lesser extent. The control group consistently outperformed the treated plants, indicating that exposure to these chemicals disrupts fundamental physiological pathways essential for plant growth and development. These findings underscore the need for further investigation into the mechanisms by which these chemicals interfere with chlorophyll synthesis, water retention, and metabolic regulation (**Supplementary Figure S1**).

### 3.4 Dose-dependent mortality induced by additive treatment

The mortality rates induced by different chemical treatments at varying concentrations are presented in **Supplementary Figure S2**. A clear dose-dependent toxic effect was observed across all chemicals tested, including DMSO, DMF, methyl oleate, and xylene, with mortality increasing as the concentration of each chemical increased. In contrast, the control group (water) exhibited minimal mortality, emphasizing the significant impact of chemical exposure. DMSO demonstrated a clear concentration-dependent increase in mortality. The highest mortality rate was observed at 40%, followed by 20% and 10% concentrations. Even at the lowest concentration (10%), mortality was significantly higher than that of the control group, indicating that DMSO has a strong toxic effect at all tested concentrations (**Supplementary Figure S2a**). DMF followed a similar pattern, with mortality exceeding 50% across all concentrations, reinforcing its high toxicity. Even at the lowest tested concentration (5%), mortality was considerable, suggesting that DMF poses a significant risk to organisms even at diluted levels. This result highlights the potential hazards of DMF, even at lower concentrations (**Supplementary Figure S2b**). Methyl oleate exhibited a concentration-dependent mortality trend, although its toxic effects appeared less severe than those of DMSO and DMF. Despite this, mortality rates at all concentrations, including the lowest, remained well above the control, indicating persistent toxicity. While not as potent as DMF or DMSO, methyl oleate still poses a considerable risk to biological systems (**Supplementary Figure S2c**). Xylene displayed a somewhat variable pattern, with mortality rates remaining consistently high across all concentrations, though slightly reduced at 10% compared to 20% and 40%. Regardless, mortality was significantly elevated compared to the control group, emphasizing xylene’s potent lethal effects. The consistent toxicity across concentrations suggests that xylene exerts strong toxic impacts on biological systems (**Supplementary Figure S2d**). From the results presented, it is clear that DMSO, DMF, and xylene exhibit the most severe toxic effects, with mortality rates remaining high across all tested concentrations. Methyl oleate, while still toxic, exhibited slightly lower mortality levels, suggesting a milder impact. The control group consistently showed minimal mortality, underscoring the dose-dependent toxicity of these substances and highlighting the potential ecological and environmental risks posed by these chemicals (additives). These findings underscore the importance of evaluating and regulating chemical exposure levels in biological systems to mitigate potential harm (**Supplementary Figure S2**).

### 3.5 Dose-dependent mortality induced by chemical insecticides

The mortality response to increasing concentrations of azadirachtin, chlorantraniliprole, and uniconazole is presented in **Supplementary Figure S3**. A clear dose-dependent trend was observed across all treatments, where increasing concentrations resulted in significantly higher mortality rates compared to the control group, which exhibited minimal mortality. Azadirachtin exhibited strong toxic effects, with mortality exceeding 80% at the highest concentration (40 mg/L). As the concentration decreased to 20 mg/L and 10 mg/L, mortality rates gradually declined but remained significantly elevated compared to the control. This suggests that azadirachtin has potent toxic effects, even at lower concentrations, highlighting the importance of careful dosage regulation (**Supplementary Figure S3a**). Chlorantraniliprole exhibited even more severe toxicity, with mortality exceeding 85% at the highest concentration (600 mg/L) and remaining consistently high at 300 mg/L and 150 mg/L. Unlike azadirachtin, mortality rates remained elevated across all tested concentrations, suggesting that chlorantraniliprole’s toxicity persists at lower doses. This may be attributed to the chemical’s mode of action, which potentially has a longer-lasting impact on survival (**Supplementary Figure S3b**). Uniconazole also induced significant mortality, with the highest concentration (15 mg/L) resulting in mortality rates above 60%. Even at lower concentrations (3 mg/L and 0.6 mg/L), mortality remained consistently elevated compared to the control. This indicates that while uniconazole is primarily a growth regulator, it exerts toxic effects when applied at higher concentrations, emphasizing the need for cautious usage (**Supplementary Figure S3c**). The findings from these experiments emphasize the importance of careful dosage regulation and toxicity assessment when using chemicals such as azadirachtin, chlorantraniliprole, and uniconazole. The observed dose-dependent mortality highlights the potential ecological risks associated with higher concentrations, underscoring the need for further research into the mechanisms of toxicity and safe application thresholds for these chemicals (**Supplementary Figure S3**).

### 3.6 Quantitative analysis of detoxification enzymes

**Figure 3:**
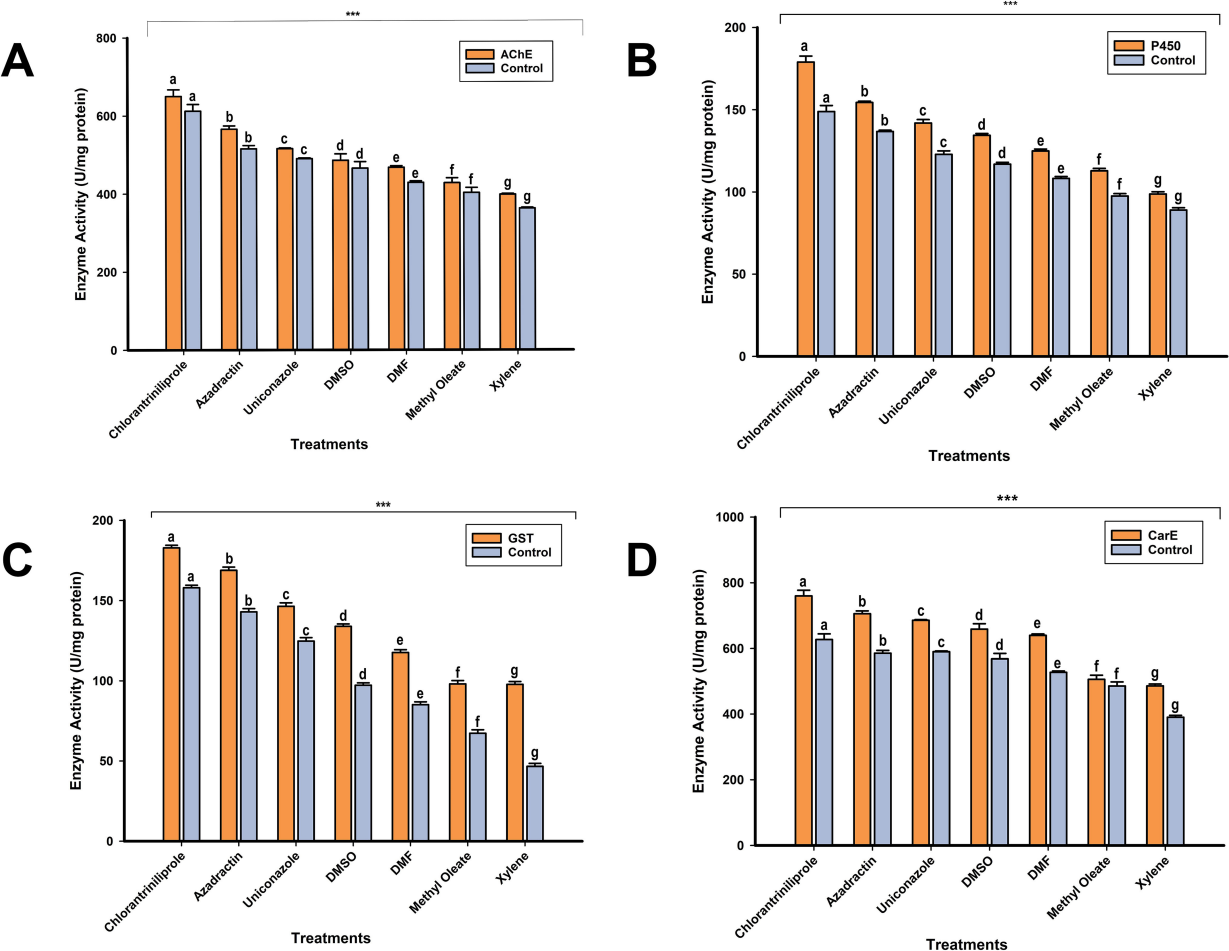
The enzyme activities of **(A)** acetylcholinesterase (AChE), **(B)** carboxylesterase (CarE), **(C)** glutathione S-transferase (GST), and **(D)** cytochrome P450 (P450) were assessed following treatment with three chemicals and four additives. Each bar represents the mean value of three replicates, with error bars indicating the standard error of the mean (SEM). Bars that are labeled with different letters denote significant differences in enzyme activity between treatments (P < 0.05, Tukey's HSD test).

**Figure 4:**
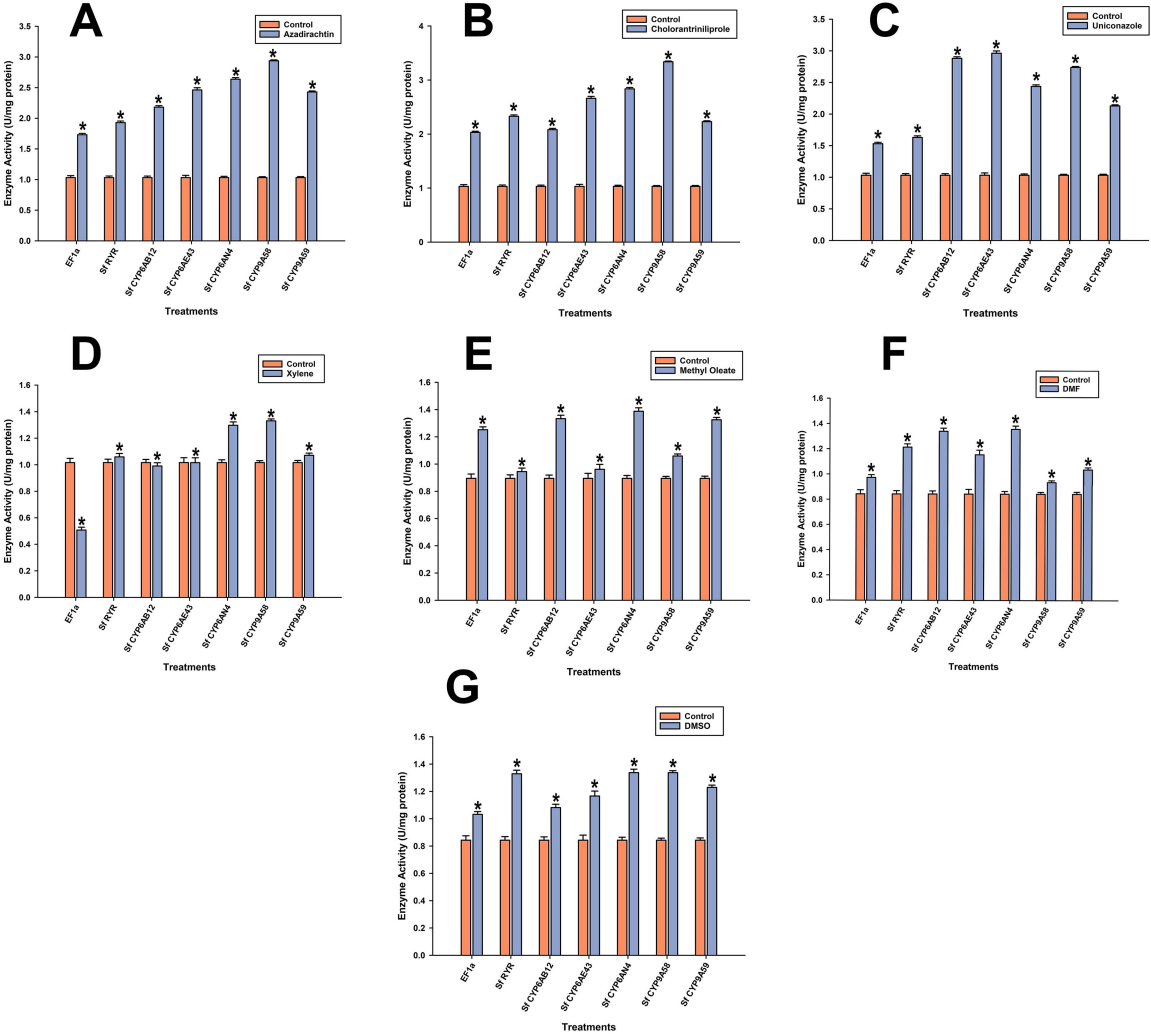
The expression levels of various insecticide resistance genes were measured following the application of three chemicals **[(A)**: Azadirachtin, **(B)**: Chlorantraniliprole, **(C)**: Uniconazole] and four additives **[(D)**: Xylene, **(E)**: Methyl Oleate, **(F)**: DMF, **(G)**: DMSO]. Gene expression was quantified using qRT-PCR, with data presented as the mean of three replicates. Error bars represent the standard error of the mean (SEM). Significant differences in gene expression between treatments are indicated by different letters (P < 0.05, Tukey's HSD test).

To investigate the role of four key detoxification enzymes in resistance development of *S. frugiperda* to different chemicals (Chlorantraniliprole, Azadirachtin, Uniconazole, DMSO, DMF, Methyl Oleate, Xylene), activities of AchE, CarEs, GSTs and P450s were analyzed. The results in **Figure 4** reveal that AchE activity varied markedly among treatments (p 0.05), but no significant difference existed between the treatments, indicating a steady participation in resistance. The GST activity was also sustained (between 0.106 and 0.166 mmol/min·mg protein, p > 0.05), and may have played minor roles in resistance mechanisms in the present study. Significant differences in cytochrome P450 between chlorantraniliprole and other treatments (0.629 nmol/min·mg protein vs. 0.273 nmol/min·mg protein) (p < 0.05) suggested that P450s played a critical role in resistance. A little difference was found in P450 activity in between supplementation and chlorantraniliprole treatment (0.439 nmol/min·mg protein) from the control group (p < 0.05). The dose-dependent mortality in chemical treatment is highly correlated with an increase in enzyme activity, especially of AchE and P450s. These enzymes are expected to be involved in the detoxification and resistance traits of *S. frugiperda*. The greater enzyme activity at higher concentration is a direct indicator of the dynamic biochemical adaptation of the insect to reduce the toxicity of the chemicals, therefore highlighting their importance in the development of resistance, as shown in **Figures 3A–D** and **Supplementary Tables 2**-**5**.

### 3.7 Olfactory response of insects to chemical (additives and insecticides) treatments in the Y-tube assay

The Y-tube olfactory bioassay was conducted to investigate the behavioral responses of insects to different chemical treatments, including DMSO, methyl oleate, DMF, xylene, uniconazole, azadirachtin, and chlorantraniliprole, across varying concentrations. The results of the analysis, summarized in **Table 1**, indicate no significant attraction or repulsion to any of the chemicals tested at the concentrations used, as suggested by the *χ*^2^ values and associated *p*-values. For DMSO, the gravid female *S. frugiperda* did not prefer the tested concentrations (10%, 20%, and 40%) against the control (hexane) in the Y-tube olfactometer. These results confirm that DMSO did not elicit a notable olfactory response at the concentrations tested. Similarly, methyl oleate also showed no significant olfactory response. The *χ*^2^ values (ranging from 0.27 to 1.67) and *p*-values further supported the lack of a significant response to the chemical at the tested concentrations. This confirms that methyl oleate did not attract or repel the insects in a statistically significant manner. The response to DMF followed a similar pattern. The *χ*^2^ values (ranging from 0.60 to 1.67) and *p*-values (0.197 to 0.979) demonstrated no significant olfactory preference, suggesting that DMF did not have an effect on the insects’ behavior across the concentrations tested. Xylene also failed to induce an insect response across all concentrations (10%, 20%, and 40%) (**Table 1**). In the case of uniconazole, azadirachtin, and chlorantraniliprole, no significant effect was observed on insect olfactory behavior. The *χ*^2^ values (≥1.67) and *p*-values (ranging from 0.197 to 0.979) indicate that none of these chemicals induced significant attraction or repulsion at the tested concentrations. The results from the Y-tube olfactory bioassays suggest that the chemicals tested (DMSO, methyl oleate, DMF, xylene, uniconazole, azadirachtin, and chlorantraniliprole) did not significantly affect insect olfactory behavior at the concentrations used (**Table 1**).

**Table 1:** Olfactory responses of gravid female *S. frugiperda* in Y-tube olfactometer bioassays.

Comparison			Insects responded		Non-responders	*χ*^2^ (*df* = 1)	*P-value*
T1		T2	T1	T2			
Dimethyl sulfoxide (DMSO) (%)	10	Hexane (Control)	29	31	9	0.07	0.796
	20	33	27	4	0.60	0.439
	40	26	34	7	1.07	0.302
Methyl oleate (%)	10	32	28	5	0.27	0.606
	20	25	35	6	1.67	0.197
	40	31	29	8	0.07	0.796
Dimethylformamide (DMF) (%)	5	25	35	4	1.67	0.197
	10	31	29	6	0.07	0.796
	20	27	33	8	0.60	0.439
Xylene (%)	10	34	26	4	1.07	0.302
	20	31	29	9	0.07	0.796
	40	27	33	6	0.60	0.439
Uniconazole (mg/L)	0.6	25	35	8	1.67	0.197
	3	33	27	7	0.60	0.439
	15	29	31	5	0.07	0.796
Azadirachtin (mg/L)	10	27	33	9	0.60	0.439
	20	31	29	8	0.07	0.796
	40	28	32	4	0.27	0.606
Chlorantraniliprole (mg/L)	150	29	31	6	0.07	0.796
300	32	28	8	0.27	0.606
600	25	35	4	1.67	0.197

### 3.8 Gene expression analysis of insecticide resistance-related genes of *S. frugiperda* in response to treatment

The expression pattern of insecticide resistance genes of *S. frugiperda* after the application of chlorantraniliprole shows significant upregulation in the treatment than the control. Specifically, genes such as (e.g., *Sf CYP6AB12, Sf CYP6AE43, Sf CYP9A58* and *Sf CYP9A59*) possessed a greater level of expression, and were good candidates for detoxification during chlorantraniliprole treatment. In contrast, *SfRYR* and *SfCYP6AN4* showed little difference in expression, suggesting a stable or weakly responsive expression profile for these genes. At large, chlorantraniliprole preferentially induced certain cytochrome P450 genes, and may be involved in insecticide resistance. The differential gene expression in response to Azadirachtin treatment shows a substantial increase in expression of a large group of detoxification-related genes in comparison with the control. Increased expressions of *SfCYP6AB12, SfCYP6AE43, and SfCYP6AN4* were observed, indicating that they played roles in detoxification function. Accelerated expression of *SfCYP9A58* and *SfCYP9A59* was slightly lower in addition, but not significantly. Remarkably, even *EF1α* and *SfRYR* were of higher abundance in the Azadirachtin–treated group, possibly reflecting how the insect is also responding in a generalist way, as it sharpens the transcriptional armamentarium to the challenge, possibly correlated to detoxification and resistance-like mechanisms. After the application of Uniconazole treatment, gene expression analysis, the majority of genes were upregulated, and their expression level increased most significantly in *SfCYP6AE43* and *SfCYP9A59*. It can be inferred that Uniconazole treatment may induce the expression of some detoxification-related genes of *S. frugiperda*. However, since the error bars indicate that gene expression varies within each group, additional statistical data are required to confirm that these expression changes are significant.

In the context of DMSO exposure, the expression of *SfCYP6AB12*, *SfCYP6AE43, SfCYP6AN4, SfCYP9A58*, and *SfCYP9A59* is significantly induced with respect to the respective control. However, the expression of *EF1α* and *SfRYR* was unchanged, suggesting that the effect of DMSO was related to the detoxification-related cytochrome P450 genes, rather than housekeeping genes. These findings indicate that DMSO may preferentially stimulate some detoxification enzymes related to metabolic resistance. Of these, the *SfCYP6AB12*, *SfCYP6AN4*, *SfCYP9A58*, and *SfCYP9A59* genes were shown to be overexpressed significantly in the DMF-treated group. However, we did not observe significant changes in *EF1α* and *SfRYR*, implying that the influence of DMF was limited to particular cytochrome P450 genes involved in detoxification, rather than affecting a large-scale transcription of genes in the sexes. The data, expressed in mean ± S.D., confirms that DMF induces some detoxification pathways in *S. frugiperda*. The gene expression profiling after the treatment with methyl oleate led to an enhanced expression of several cytochrome P450 genes like *SfCYP6AB12*, *SfCYP6AE43*, *SfCYP6AN4*, *SfCYP9A5A8*, and *SfCYP9A5A9* when compared with the control. This may imply that there is induction of certain cytochrome P450 enzymes that participate in the detoxification of methyl oleate. In contrast, other candidate internal control genes *EF1α* and *SfRYR* remained constant between treatment and control groups, suggesting that methyl oleate might result in a detoxification-related gene-specific effect. It is interesting to note that several cytochromes P450 genes (e.g., *SfCYP6AB12, SfCYP6E43, SfCYP6AN4, SfCYP9A5B*, and *SfCYP9A9*) were significantly (marked by asterisks in the Figure) upregulated after exposure to Xylene. In contrast, for *EF1α* and *SfRYR* there were no observed differences, indicating Xylene treatment does not influence these gene expressions. These data suggest a selective activation of the detoxification cytochrome P450 genes in response to Xylene, strengthening the role of these gene families in the metabolic adaptive response of the insect to environmental challenges, as shown in **Figures 4A–G**.

## 4 Discussion

*S. frugiperda* is an invasive pest; not only does the unscientific use of chemical insecticides in the field result in the outbreak of resistance, but invasive pests themselves may also carry resistance to certain insecticides (Gao et al., 2013). This study underscores the significant role of various chemicals, including DMSO, methyl oleate, DMF, xylene, uniconazole, azadirachtin, and chlorantraniliprole, in modulating both the physiological responses of *S. frugiperda* and the photosynthetic efficiency of maize. Our results align with those of Shenashen et al. (2017), who demonstrated that lower concentrations of these chemicals enhance photosynthetic efficiency, while higher concentrations reduce photosynthetic rates. This concentration-dependent effect on photosynthesis corroborates findings in earlier studies that report varied responses in herbivore-induced changes in assimilation rates, with some studies indicating increases in assimilation (Oleksyn et al., 1998; Nykänen and Koricheva, 2004) and others showing decreases (Nabity et al., 2009). Our study emphasizes that the photosynthetic response is influenced by the chemical concentration, highlighting the importance of precise dosage to optimize plant physiological responses. The resistance of *S. frugiperda* to chlorantraniliprole in our study was primarily driven by the upregulation of detoxification enzymes, especially cytochrome P450s (P450s), which is consistent with the findings of Gong et al. (2013) and Elzaki et al. (2016), who reported that overexpression of specific P450 genes contributes to resistance. Our enzyme activity analysis demonstrated that chlorantraniliprole treatment significantly increased P450 activity (0.629 nmol/min·mg protein), compared to other treatments (0.273 nmol/min·mg protein). This supports the critical role of P450s in chlorantraniliprole resistance in *S. frugiperda*, consistent with studies by Xu et al. (2019) and Tan et al. (2022), which suggest that P450s are key players in the metabolic resistance of *S. frugiperda* to insecticides. Conversely, AchE and GST activities showed no significant differences between treatments, suggesting that these enzymes have a minimal role in resistance in this context, which contrasts with some previous studies (Zhang et al., 2020).

Our Y-tube olfactometer assay did not reveal significant changes in olfactory behavior in response to chemicals such as DMSO, Methyl Oleate, DMF, Xylene, Uniconazole, Azadirachtin, and Chlorantraniliprole, indicating that these treatments did not alter attraction or repulsion behavior. This contrasts with earlier studies (Brilli et al., 2019; Turlings and Erb, 2018), which reported that similar chemicals could influence pest populations through behavioral mechanisms. The lack of significant behavioral changes in our study suggests that the observed pest mortality is more likely due to direct chemical toxicity or physiological disruption rather than behavioral alterations. This supports the notion that the efficacy of these chemicals in pest control stems primarily from their direct physiological effects on the pest, rather than behavioral modifications.

The differential expression of cytochrome P450 genes in response to various treatments further elucidates their crucial role in the detoxification and resistance mechanisms of *S. frugiperda*. Specifically, exposure to chlorantraniliprole significantly upregulated genes such as *SfCYP6AB12, SfCYP6AE43, SfCYP9A58*, and *SfCYP9A59*, suggesting their potential involvement in insecticide resistance. These findings are consistent with previous studies by Gong et al. (2013) and Elzaki et al. (2016), which indicated that overexpression of specific P450 genes, such as *CYP305A* and *CYP49A1*, is strongly correlated with chlorantraniliprole resistance. Furthermore, gene expression profiles after treatment with Azadirachtin, Uniconazole, DMSO, DMF, Methyl Oleate, and Xylene revealed the selective induction of detoxification-related cytochrome P450 genes, particularly *SfCYP6AB12, SfCYP6AN4*, and *SfCYP9A58*. These findings suggest that these enzymes play a pivotal role in the metabolic resistance of *S. frugiperda*, highlighting the activation of specific cytochrome P450 genes as a primary mechanism of insecticide resistance. The differential induction of these enzymes by different environmental chemicals further emphasizes the adaptability of *S. frugiperda* to insecticides.

## 5 Conclusion

In summary, the present report demonstrates that both maize photosynthesis and *S. frugiperda* mortality were concentration-dependent for chemical treatment. The results reveal that the cytochrome P450 superfamily may play a crucial part in the insecticide resistance of the insect, of which *SfCYP6AB12, SfCYP6AE43, SfCYP9A58*, and *SfCYP9A59* have been upregulated significantly with chlorantraniliprole and other chemicals. No substantive alterations in behavior were produced, and mortality was dominated by direct chemical toxic effects. These findings highlight the necessity to elucidate the molecular resistance mechanisms, as well as to tightly control chemical application for pest management.

## Data Availability

The original contributions presented in the study are included in the article/**Supplementary Material**. Further inquiries can be directed to the corresponding authors.
