# Supplementary material for: The impact of chemicals and additives on maize physiology and resistance to Spodoptera frugiperda
Source: Front Plant Sci. 2026 Jan 28;17:1767144. doi: 10.3389/fpls.2026.1767144 (PMC12891130; doi:10.3389/fpls.2026.1767144)
Supplement: Supplementary file 1 [file DataSheet1.docx]

**Supplementary File:**

**Table S1: Primer used for qRTPCR:**

| **Gene Accession**  **Number** | **Gene Name** | **Forward primer 5’-- 3’** | **Reverse Primer 5’-- 3’** |
| --- | --- | --- | --- |
| **U20139.1** | EF1α | ATGGATCAACAATGGGCGA | GCCAGCAGAAATCTCCGTC |
| **MK226188.1** | Sf RYR | TCCCAGTGGATCTCCACAGG | CAACGTACACCTTGCGTCGA |
| **KC789747.1** | Sf CYP6AB12 | TACTGGCTGCGGTCCGCTATA | CTACTTGCCAGCTCGTTACTG |
| **KJ671575.1** | Sf CYP6AE43 | TACTTCGGCACGTTGGAGCT | GTCGACACTTTCCCAAGCG |
| **KC789748.1** | Sf CYP6AN4 | ATTTACGACGCCAACGCTTGC | CAATTCACCCAATCTCTGTCTG |
| **KJ671577.1** | Sf CYP9A58 | CGAGGCAGTGCTTTCATGAA | GAGCGCATACTTAGTGGGTG |
| **KJ671578.1** | Sf CYP9A59 | CGACCCAGAACTGATTAGTACG | CTCTTTCATTTCCTCGTCCACAT |

**Table S2: ANNOVA table of AChE Enzyme:**

| **Source** | **DF** | **SS** | **MS** | **F** | **P** |
| --- | --- | --- | --- | --- | --- |
| Treatment | 2 | 64468 | 32234.1 | 12.6 | 0.0006*** |
| Error | 15 | 38276 | 2551.7 |  |  |
| Total | 17 | 102744 |  |  |  |

**Table S3: ANNOVA table of CarE Enzyme:**

| **Source** | **DF** | **SS** | **MS** | **F** | **P** |
| --- | --- | --- | --- | --- | --- |
| Treatment | 2 | 64468 | 32234.1 | 12.6 | 0.0006*** |
| Error | 15 | 38276 | 2551.7 |  |  |
| Total | 17 | 102744 |  |  |  |

**Table S4: ANNOVA table of GST Enzyme**

| **Source** | **DF** | **SS** | **MS** | **F** | **P** |
| --- | --- | --- | --- | --- | --- |
| Treatment | 2 | 341.59 | 170.797 | 0.27 | 0.0137* |
| Error | 15 | 9332.47 | 622.165 |  |  |
| Total | 17 | 9674.07 |  |  |  |

**Table S5: ANNOVA table of P450 Enzyme**

| **Source** | **DF** | **SS** | **MS** | **F** | **P** |
| --- | --- | --- | --- | --- | --- |
| Treatment | 2 | 1949.67 | 974.833 | 3.11 | 0.0443* |
| Error | 15 | 4706.03 | 313.735 |  |  |
| Total | 17 | 6655.69 |  |  |  |

**Figure S1:**


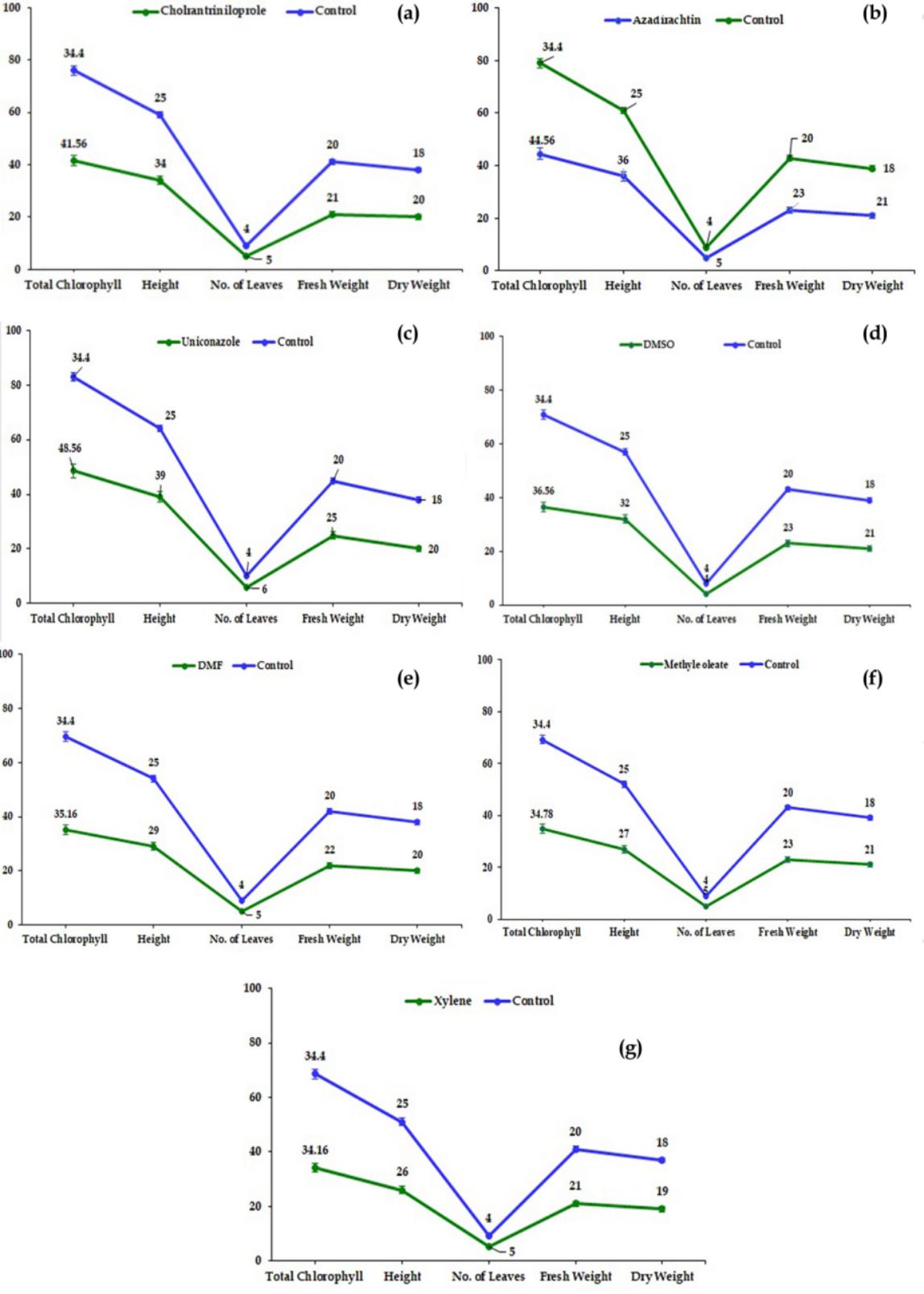


Figure S1. Impact of chemical treatments on key plant physiological traits, including **total chlorophyll content, plant height, number of leaves, fresh weight, and dry weight. (a–g)** Different chemicals [chlorantraniliprole, azadirachtin, uniconazole, dimethyl sulfoxide (DMSO), dimethyl formamide (DMF), methyl oleate, and xylene) significantly reduced growth parameters compared to the **control group**, which maintained higher values across all traits. **Xylene, uniconazole, and chlorantraniliprole**

**Figure S2:**


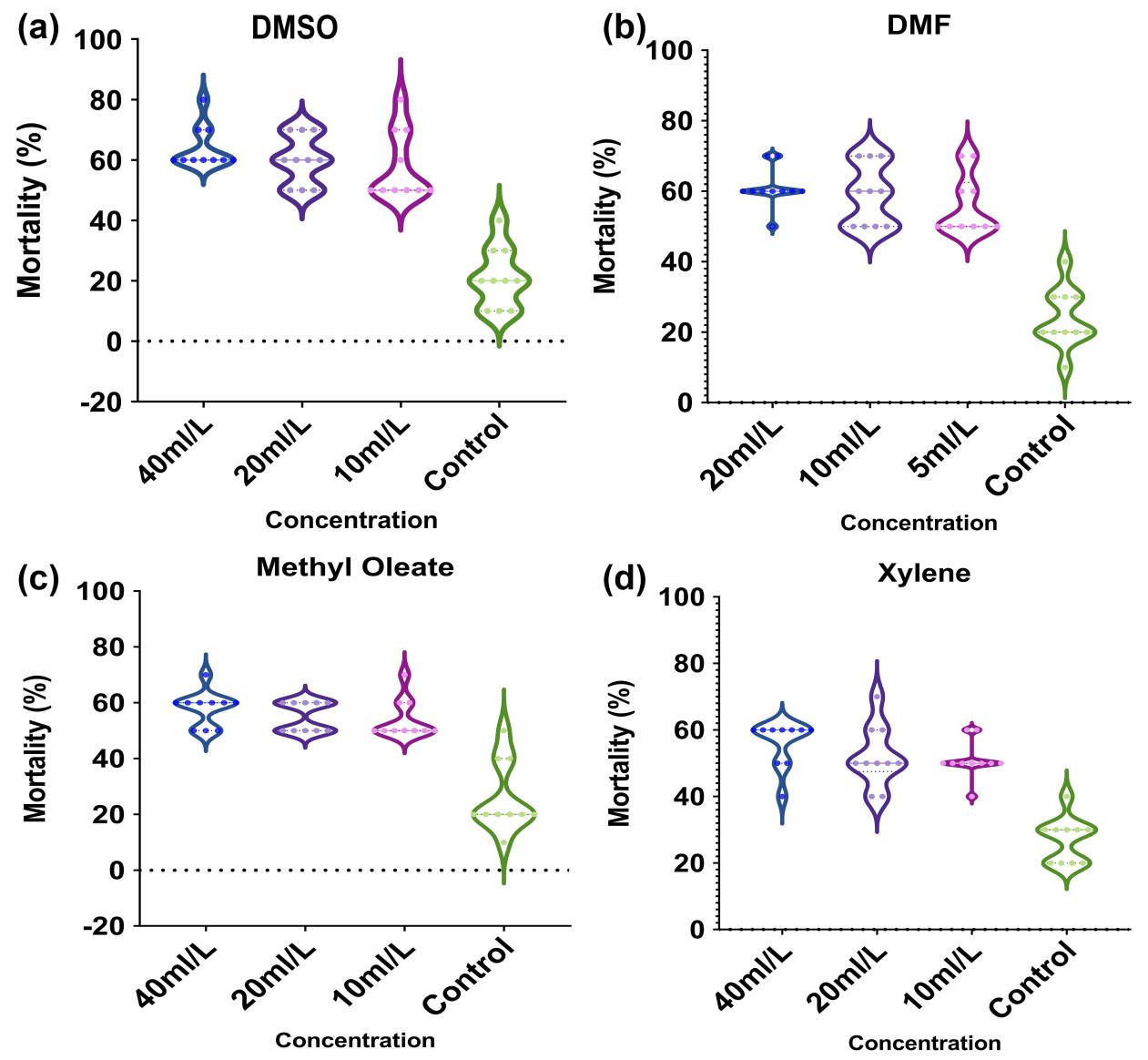


**Figure S2.** Mortality rates (%) induced by different chemical treatments at varying concentrations. **(a)** dimethyl sulfoxide (DMSO)**, (b)** dimethyl formamide (DMF)**, (c) methyl oleate, and (d) xylene** exhibit a **dose-dependent increase in toxicity,** with higher concentrations leading to greater mortality. Across all treatments, the control group showed minimal mortality, confirming the significant impact of chemical exposure. **DMF and DMSO displayed the highest toxicity**, while **methyl oleate had a slightly lower but still notable effect.** Violin plots represent data distribution, with individual data points and mean mortality values.

**Figure S3:**


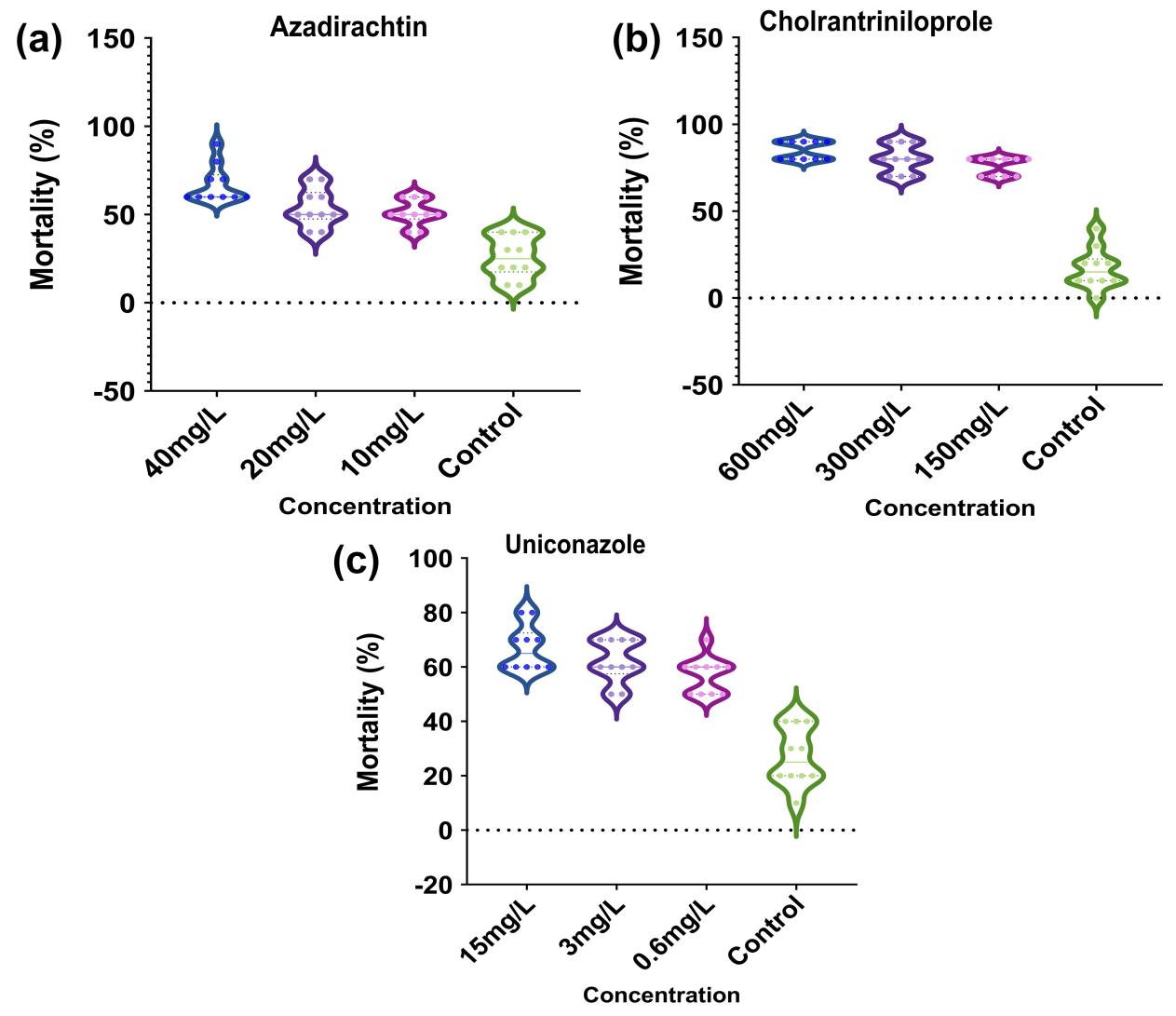


**Figure S3.** Mortality rates (%) in response to different concentrations of **(a) azadirachtin, (b) chlorantraniliprole, and (c) uniconazole**. A **dose-dependent increase in mortality** is observed across all treatments, with higher concentrations leading to significantly elevated mortality rates compared to the **control group**, which exhibited minimal mortality. **Chlorantraniliprole demonstrated the highest toxicity**, maintaining consistently high mortality across all concentrations, while **azadirachtin and uniconazole exhibited strong but slightly more concentration-dependent effects**. Violin plots show data distribution, with individual data points and mean mortality values.
